# Pathogenic bacteria associated with outbreaks of respiratory disease in Iranian broiler farms

**DOI:** 10.1002/vms3.1162

**Published:** 2023-05-21

**Authors:** Mohsen Bashashati, Mohammad Shojaei, Fereshteh Sabouri

**Affiliations:** ^1^ Department of Avian Disease Research and Diagnostics Razi Vaccine and Serum Research Institute, Agricultural Research Education and Extension Organization (AREEO) Karaj Iran; ^2^ Department of Aerobic Bacterial Research and Vaccine Production Razi Vaccine & Serum Research Institute, Agricultural Research Education and Extension Organization (AREEO) Karaj Iran

**Keywords:** bacterial respiratory pathogens, multiple infections, *Mycoplasma gallisepticum*, *Mycoplasma synoviae*, *Ornithobacterium rhinotracheale*, poultry

## Abstract

**Background:**

Multi‐causal respiratory infections are more commonly observed than uncomplicated cases with single agents in the commercial poultry industry. Recently, increased mortality rates associated with respiratory clinical signs have been reported in Iranian broiler farms.

**Objectives:**

The present study aimed to determine the spectra of avian mycoplasmas (*Mycoplasma gallisepticum*, MG and *Mycoplasma synoviae*, MS) and *Ornithobacterium rhinotracheale* (ORT) in the broiler farms with the multi‐causal respiratory disease (MCRD) from 2017 to 2020.

**Methods:**

Trachea and lung tissue samples were collected from 70 broiler flocks presenting increased mortality and acute respiratory disease. MG, MS, and ORT were detected by performing polymerase chain reaction with primers complementary to the 16S rRNA, *vlhA*, and 16S rRNA genes, respectively.

**Results:**

Genetic materials of MG, MS, and ORT were detected in five, three, and five of the 70 flocks. Based on the phylogenetic analysis of the complete *mgc2* coding sequences, all MG strains formed a distinct cluster along with other Iranian MG isolates. According to the phylogenetic analysis of the partial *vlhA* gene of MS strains, two isolates were located along with Australian and European strains. In addition, one of them displayed an out‐group association with MS isolates from Jordan. Phylogenetic analysis of Iranian ORT strains using a partial sequence of the 16S rRNA gene showed a distinct group among the other ORT strains.

**Conclusions:**

The results indicate that MG, MS, and ORT are not predominantly responsible for the MCRD. However, continuous monitoring of poultry flocks could be significant for obtaining valuable information related to different MG, MS, and ORT strains and designing effective control strategies.

## Graphical abstract

Phylogenetic relationships among MG, MS and ORT strains based on the partial sequence of the *mgc2*, *vlhA*, and 16S rRNA genes, respectively. The trees were generated by the neighbor‐joining method using MEGA X software. The sequences from this study are indicated by the triangle shaped symbol.

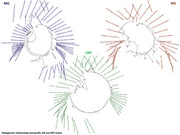

## 1 INTRODUCTION

The high prevalence of the multi‐causal respiratory disease (MCRD) in broiler flocks has recently caused heavy economic losses in Iran. Viral pathogens such as *Avian orthoavulavirus 1* (AOaV‐1), *Avian coronavirus* (ACV), *Alphainfluenzavirus* (AIV), and *Avian metapneumovirus* (AMPV) are considered among the major causes of concomitant respiratory infections. In addition to these viruses, bacterial agents such as *Mycoplasma gallisepticum* (MG), *Mycoplasma synoviae* (MS), and *Ornithobacterium rhinotracheale* (ORT) are involved in exacerbating the disease. Furthermore, respiratory reactions resulting from routine vaccination programs may play a significant role in developing respiratory diseases. The impact of the MCRD depends on the virulence of infecting pathogens, age of birds, immunosuppressive agents, environmental conditions, and the vaccine reaction (Pantin‐Jackwood & Spackman, 2020).

The most common multi‐causal respiratory infections are related to avian pathogenic mycoplasmas especially MG and MS. MG causes chronic respiratory disease in chickens and infectious sinusitis in turkeys (Pantin‐Jackwood & Spackman, 2020). This mycoplasma can cause various clinical signs such as respiratory distress, nasal discharge, sinusitis, airsacculitis, decreased egg production, and increased embryonic mortality. Poor weight gain, high feed conversion ratio, increased carcass condemnation rate, and mortality have been reported in broilers. In addition to MG‐like respiratory clinical signs, MS leads to eggshell apex abnormalities and egg production losses. Furthermore, arthropathic‐ and amyloid‐inducing strains may cause severe economic losses due to the reduced growth rate and lameness. Although infection with MG and MS may go unnoticed clinically, birds may be more susceptible to secondary infections with other bacteria such as *Escherichia coli* and viruses such as AOaV‐1, ACV, and AIV. The control of MG and MS can be achieved through adaptation of strict biosecurity, vaccination, and medication. Because MG and MS can be transmitted vertically, eradication of MG and MS in infected breeder flocks is the main approach of mycoplasmosis prevention and control (Ferguson‐Noel, 2020). Currently, the most important method for the control of MG in Iranian breeder flocks is biosurveillance and voluntary elimination of infected flocks. In addition to improvement of biosecurity, control measure for MS in Iran includes vaccination of breeder flocks using the MS‐H vaccine (Bashashati & Banani, 2020). Vaccination plays an essential role in preventing/reducing respiratory disease and protecting against egg production drops (Ferguson‐Noel, 2020).

ORT can play a primary or secondary role in a variety of respiratory infections of chickens and turkeys depending on strain pathogenicity, environmental factors, host immune status, and the presence of other infectious agents. Severe economic losses due to high mortality and condemnation rates, decreased egg production, and reduced growth rate may be associated with ORT infection. The severity of clinical signs, disease duration, and losses of ORT outbreaks are highly variable and are affected by numerous environmental conditions such as poor management, inadequate ventilation, high density, poor‐quality bedding, poor hygiene, high levels of ammonia, concomitant disease, and type of secondary infection. Clinical signs in broiler chickens include listlessness, reduced mobility, decreased feed intake, weight loss, transient nasal discharge, sneezing, and facial oedema. In broiler breeders, the disease affects the birds in the laying period, especially the period of peak production or prior to start of egg production. In addition, it is associated with an increase in mortality, reduced feed intake, and mild respiratory clinical signs. On the other hand, it may decrease egg production, reduce egg size, and lead to poor shell quality in the flocks. In laying hens, decreased egg production, increased misshapen eggs, and deaths associated with ORT infection have been reported (Hafez & Chin, 2020). ORT control is primarily performed through strict biosecurity methods to prevent infection from spreading among the flock. Vaccination of breeders by inactivated vaccine stimulates the production of high levels of antibodies that can pass to their progeny to protect them for up to 4 weeks of age (Warner et al., 2009).

Three main approaches could be used for the diagnosis of MG, MS, and ORT including isolation and identification, serology, and molecular detection (Armour, 2020; Ferguson‐Noel & Noormohammadi, 2020; Hafez & Chin, 2020). Although the bacterial culture approach is considered the gold standard, this technique is tedious and time‐consuming, especially for avian mycoplasmas, and may require up to 4 weeks. In addition, culture methods become difficult in samples infected with other commensal mycoplasmas and fast‐growing bacteria or those with a history of previous antibiotic therapy. Routine serological techniques to detect avian pathogenic mycoplasmas are the rapid slide agglutination (RSA), enzyme‐linked immunosorbent assay (ELISA), and haemagglutination inhibition (HI) tests. The RSA and ELISA methods are used for serologic evaluation of ORT infection in the poultry industry. Although serologic procedures have advantages over bacterial isolation (e.g., antibodies are detectable for several weeks after infection), they have drawbacks because of the nonspecific reactions and the interval between infection and appearance of adaptive immunity. In addition, polymerase chain reaction (PCR) and quantitative PCR have been designed as fast, sensitive, and specific molecular methods to detect suspicious isolates (Armour 2020; Ferguson‐Noel & Noormohammadi, 2020; Hafez & Chin, 2020).

Under commercial field conditions, co‐infection with multiple respiratory pathogens is more common than single respiratory infection. Despite the various studies on single MG, MS, and ORT infections in Iran, these bacteria have not been studied together, and their contribution to the MCRD has not been determined (Banani et al., 2004; Bashashati & Banani, 2020; Bayatzadeh et al., 2014). Due to the high prevalence of MCRD in the Iranian broiler flocks and understanding of the possible involvement of bacterial pathogens in the development of multiple respiratory infections, this study was aimed at molecular detection and phylogenetic analysis of avian pathogenic mycoplasmas (MG and MS) and ORT in recent outbreaks. Moreover, among the PCR‐positive tissue samples, reverse transcription‐PCR (RT‐PCR) was performed to detect common viral respiratory pathogens (AOaV‐1, ACV, and AIV subtype H9N2) in order to determine whether the poultry with clinical signs are coinfected with these avian viruses.

## 2 MATERIALS AND METHODS

### 2.1 Clinical information, sample collection, and processing

For this study, 350 specimens including tracheal and lung tissues from a total of 70 broiler farms (5000—80,000 birds per flock) were collected in 12 provinces of Iran (i.e., Alborz, Bushehr, Chaharmahal and Bakhtiari, Hamadan, Hormozgan, Isfahan, Kermanshah, Markazi, Mazandaran, Tehran, West Azerbaijan, and Yazd) between 2017 and 2020. The collected samples were not a routine and systematic monitoring, but they were originated from broilers at 3–6 weeks old displaying the overt clinical respiratory signs with high rate of mortality for a minimum of 3 days, which required a further laboratory diagnosis. Tissue samples (trachea and lungs) from five birds of each flock were pooled together and homogenized in sterile mortar and pestle with brain heart infusion broth to get 10% concentration (w/v). After centrifugation at 1000 × *g* for 5 min, the supernatant was stored at −70°C until use.

### 2.2 Genomic DNA extraction

DNA was extracted by the phenol‐chloroform method with some modifications (Ghadersohi et al., 1997). Briefly, 1 mL of the supernatant was transferred to a 1.5 mL microtube and centrifuged at 13,000 × *g* for 30 min. 900 μL of supernatant was discarded, and 100 μL of lysis buffer (50 mM Tris‐HCl‐50 mM EDTA‐100 mM NaCl and 10% SDS) was added. After mixing, 100 μg/mL proteinase K was added to the lysate, and the mixture was incubated at 56°C for 4 h. The mixture was subjected to three extractions with tris‐saturated phenol (pH 8.0), phenol‐chloroform‐isoamyl alcohol (25:24:1), and chloroform‐isoamyl alcohol (24:1) until a clean aqueous phase was obtained. 0.1 volume of sodium acetate buffer (3 M, pH 5.2) was added to the solution, and the DNA was precipitated with 2X sample volume of 100% ethanol. After washing the residue with ice cold 70% ethanol, DNA was diluted in 50 μL of nuclease‐free water and kept at −20°C.

### 2.3 Polymerase chain reaction

MG, MS, and ORT bacteria were detected in tissue samples using the primers shown in Table 1. These primers were designed to amplify fragments of 531 and 784 bp of 16S rRNA gene for MG and ORT, respectively, and 341–392 bp of *vlhA* gene for MS (Jeffery et al., 2007; Kiss et al., 1997; van Empel & Hafez, 1999). The same primers were used for molecular analysis of MS and ORT. Complete length of *mgc2* gene was amplified using primers MB‐*mgc2*‐F and MB‐*mgc2*‐R that were previously described (Bashashati & Banani, 2020). In the studied positive flocks, further analysis was performed to detect other respiratory pathogenic viruses (AOaV‐1, ACV, and AIV subtype H9N2) using RT‐PCR based on the previously released methods (Creelan et al., 2002; Lee et al., 2001; Loa et al., 2006; Qiu et al., 2009). The primer set used for the detection of these viruses is also shown in Table 1. PCR products were electrophoresed on a 1.5% agarose gel and stained with SYBR Safe DNA Gel‐Stain (Thermo Fisher Scientific, MA, USA).

**TABLE 1:** The sequence of primers used in this study for detection and sequencing.

Name of primers	Target gene	Sequence	PCR product (bp)	Used for	Reference
MB‐MG‐10F	16S rRNA	5′‐AACACCAGAGGCGAAGGCGAGG‐3′	531	Detection of MG	Kiss et al. (1997)
MB‐MG‐11R		5′‐ACGGATTTGCAACTGTTTGTATTGG‐3′			
MB‐Link	*vlhA*	5′‐TACTATTAGCAGCTAGTGC‐3′	341–392	Detection and sequencing of MS	Jeffery et al. (2007)
MB‐MSCons		5′‐AGTAACCGATCCGCTTAAT‐3′			
MB‐OR16S‐F1	16S rRNA	5ʹ‐GAGAATTAATTTACGGATTAAG‐3ʹ	784	Detection and Sequencing of ORT	van Empel and Hafez (1999)
MB‐OR16‐R1		5ʹ‐TTCGCTTGGTCTCCGAAGAT‐3ʹ			
MB‐*mgc2*‐F	*mgc2*	5′‐TGATTGCTATGGTGGGCTTG‐3′	1384	Sequencing of MG	Bashashati and Banani (2020)
MB‐*mgc2*‐R		5′‐ACCGATTAAGGCAAGAGGAGTT‐3′			
MB‐NDV‐F	Fusion	5′‐GGTGAGTCTATCCGGARGATACAAG‐3′	202	Detection of AOaV‐1	Creelan et al. (2002)
MB‐NDV‐R		5′‐TCATTGGTTGCRGCAATGCTCT‐3′			
MB‐N103F	Nucleocapsid	5′‐CCTGATGGTAATTTCCGTTGGG‐3′	357	Detection of ACV	Loa et al. (2006)
MB‐N102R		5′‐ACGCCCATCCTTAATACCTTCCTC‐3′			
MB‐H9‐151f	Hemagglutinin	5′‐CTYCACACAGARCACAATGG‐3′	488	Detection of AIV subtype H9	Lee et al. (2001)
MB‐H9‐638r		5′‐GTCACACTTGTTGTTGTRTC‐3′			
MB‐NA2‐1	Neuraminidase	5′‐TCCGTTTCATTTGGGAACC‐3′	314	Detection of AIV subtype N2	Qiu et al. (2009)
MB‐NA2‐2		5′‐CTGACAATGGRCTAATGTG‐3′			

Abbreviations: ACV, *A* *vian coronavirus*; AIV, *Alphainfluenzavirus*; AOaV‐1, *Avian orthoavulavirus‐1*; MG, *Mycoplasma gallisepticum*; MS, *Mycoplasma synoviae*; ORT, *Ornithobacterium rhinotracheale*; PCR, polymerase chain reaction.

### 2.4 Genomic sequencing

The amplicons obtained using PCR were excised from the agarose gel and purified using the GeneJET Gel extraction Kit (Thermo Fisher Scientific). The purified PCR products were cloned in the pJET1.2/blunt vector (Thermo Fisher Scientific) according to the manufacturer's instructions, and these were used to transform *E. coli* TOP10 competent cells. Bacterial colonies were selected and checked for the integrated desired PCR product by PCR, as well as restriction endonuclease digestion using *bgl*II (Thermo Fisher Scientific). Positive clones were cultured in LB broth containing 100 μL/mL ampicillin, and the plasmid was extracted using the alkaline lysis method (Feliciello & Chinali, 1993). Recombinant plasmids with target genes were sequenced in both directions: vector forward and reverse primers. Sequencing services were provided by Macrogen, Seoul, South Korea.

### 2.5 Nucleotide sequencing and phylogenetic study

Nucleotide sequence editing, analysis, and alignments were performed using the BioEdit program (version 7.1.3.0) (Hall, 1999). Nucleotide sequences were compared with corresponding sequences available in the GenBank database using BLAST analysis (https://blast.ncbi.nlm.nih.gov/Blast.cgi). The phylogenetic trees were constructed using the neighbour‐joining method with maximum composite likelihood model in the MEGA software (version X) for *mgc2*, *vlhA*, and 16S rRNA genes (Kumar et al., 2018). The topological accuracy of the trees was evaluated by the bootstrap method with 1000 replications.

## 3 RESULTS

### 3.1 Molecular detection of MG, MS, and ORT, and other viruses involved in the MCRD

Table 2 shows the concomitant respiratory pathogens identified in broiler chicken flocks suffering from respiratory disease. The studied bacteria were detected in flocks with the MCRD from the broilers in Isfahan, Kermanshah, Khorasan Razavi, Mazandaran, Tehran, and Yazd provinces. Out of total 70 flocks, five, three, five were found to be positive for MG, MS, and ORT, respectively. Among the nine positive flocks for MG, MS, and ORT, other viral respiratory pathogens involved in the MCRD were detected to determine the probable role of viral infections in the development of clinical signs. Based on the obtained results, eight flocks were infected with AOaV‐1, ACV, and AIV subtype H9N2 (Table 2).

**TABLE 2:** Broiler flocks positive for different respiratory pathogens.

Respiratory pathogens^a^							
MG	MS	ORT	AIV subtype H9N2	ACV	AOaV‐1	Location	Time
MG‐MCRD‐1	‐	‐	‐	**✓**	‐	Isfahan	Dec. 2017
‐	MS‐MCRD‐1	‐	**✓**	‐	‐	Mazandaran	Dec. 2017
MG‐MCRD‐2	‐	ORT‐MCRD‐1	**✓**	**✓**	‐	Yazd	Jan. 2018
MG‐MCRD‐3	‐	ORT‐MCRD‐2	‐	‐	‐	Yazd	Mar. 2018
‐	MS‐MCRD‐2	‐	‐	‐	**✓**	Yazd	Mar. 2018
‐	MS‐MCRD‐3	ORT‐MCRD‐3	‐	‐	**✓**	Yazd	Mar. 2018
MG‐MCRD‐4	‐	ORT‐MCRD‐4	‐	‐	**✓**	Khorasan Razavi	Nov. 2018
‐	‐	ORT‐MCRD‐5	**✓**	‐	‐	Kermanshah	Mar. 2019
MG‐MCRD‐5	‐	‐	‐	‐	**✓**	Tehran	Apr. 2020

Abbreviations: ACV, *A* *vian coronavirus*; AIV, *Alphainfluenzavirus*; AOaV‐1, *Avian orthoavulavirus‐1*; MG, *Mycoplasma gallisepticum*; MS, *Mycoplasma synoviae*; ORT, *Ornithobacterium rhinotracheale*; PCR, polymerase chain reaction.

^a^ Number of tested flocks: 70 flocks; all were suffering from respiratory disease.

### 3.2 Sequence comparison and phylogenetic analysis

The recombinant plasmids were purified through alkaline‐lysis method and commercially Sanger sequenced. The genetic comparison of nucleotide and amino acid sequences of the *mgc2* genes showed that five MG strains were found to share 100% similarity with each other. The homologies of nucleotide and amino acid sequences observed between the studied *vlhA* genes were 84.7%–100% and 85.1%–100%, respectively. The two studied MS strains possessed 100% similarity at nucleotide and amino acid levels, while one of the isolates from Mazandaran province (MS‐MCRD‐1) had a unique sequence. Among the studied ORT strains, nucleotide sequences of the 16S rRNA genes showed 100% similarity.

**FIGURE 1:**
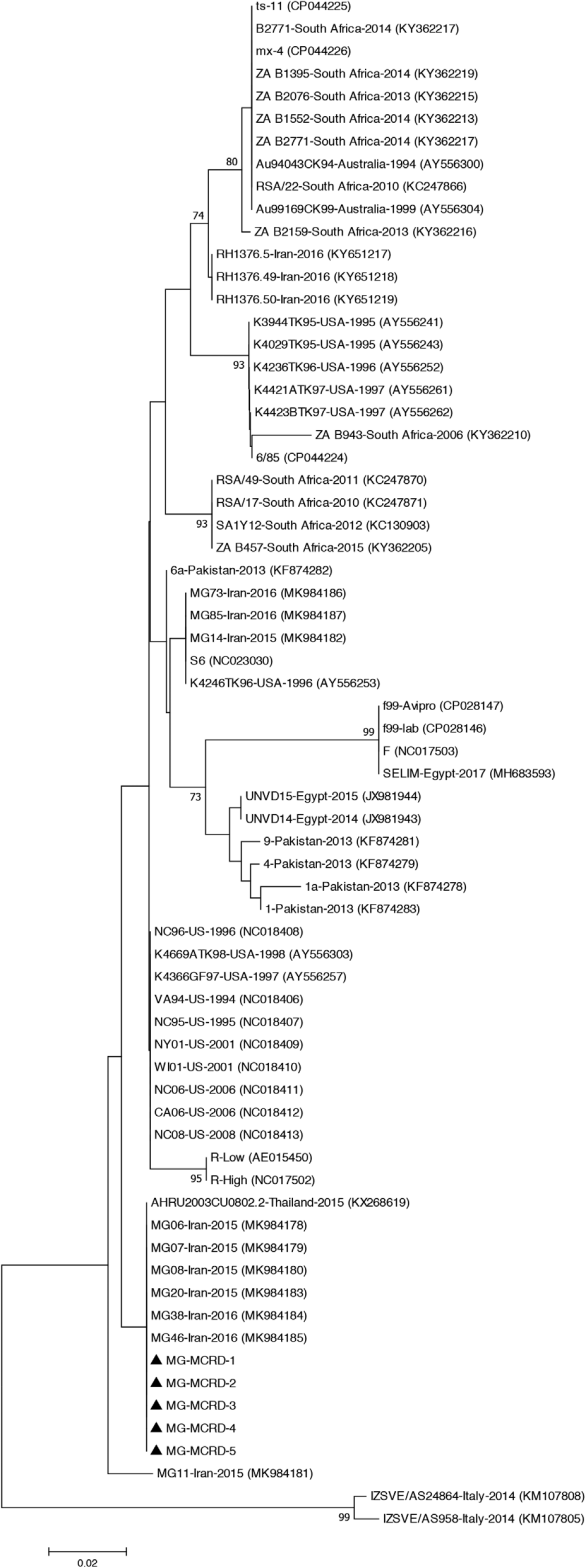
Phylogenetic relationships of the nucleotide sequences of *Mycoplasma gallisepticum* (MG) strains based on the partial sequence of the *mgc2* gene. The tree was generated by the neighbour‐joining method using the MEGA X software. The sequences from this study are indicated by the triangle‐shaped symbol.

**FIGURE 2:**
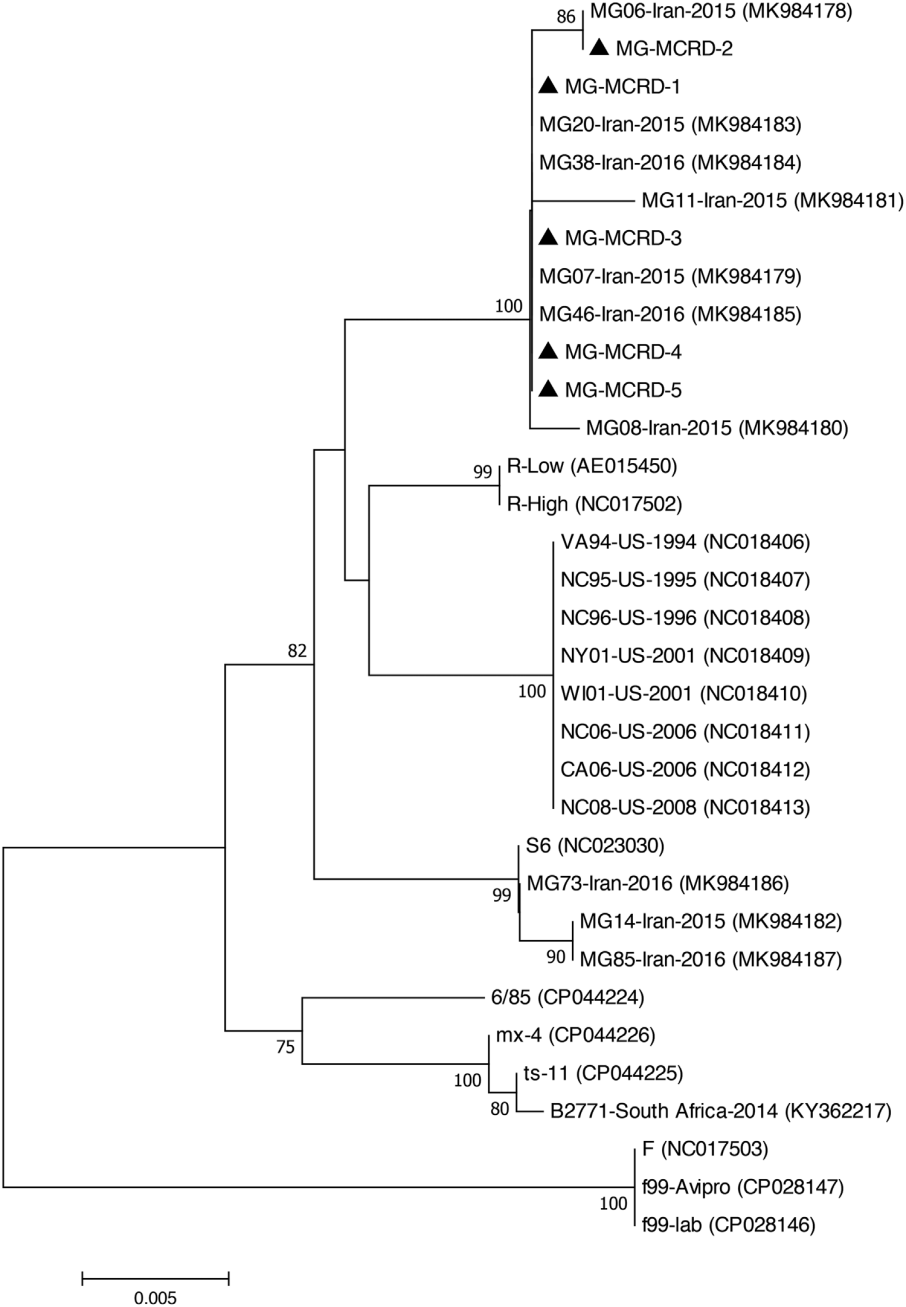
Phylogenetic relationships of the nucleotide sequences of *Mycoplasma gallisepticum* (MG) strains based on the complete coding sequence of the *mgc2* gene. The tree was generated by the neighbour‐joining method using the MEGA X software. The sequences from this study are indicated by the triangle‐shaped symbol.

**FIGURE 3:**
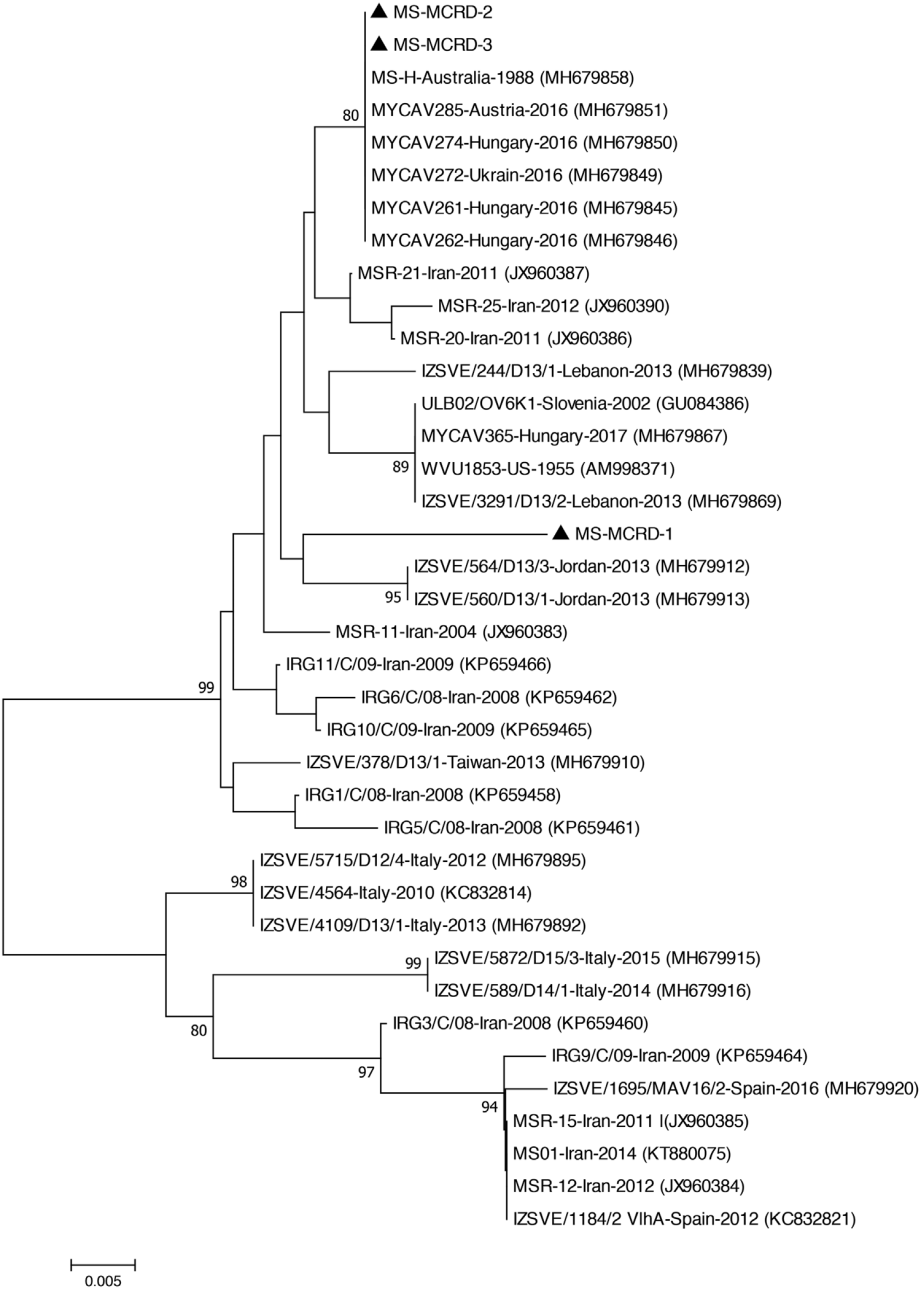
Phylogenetic relationships of the nucleotide sequences of *Mycoplasma synoviae* (MS) strains based on the partial sequence of the *vlhA* gene. The tree was generated by the neighbour‐joining method using the MEGA X software. The sequences from this study are indicated by the triangle‐shaped symbol.

**FIGURE 4:**
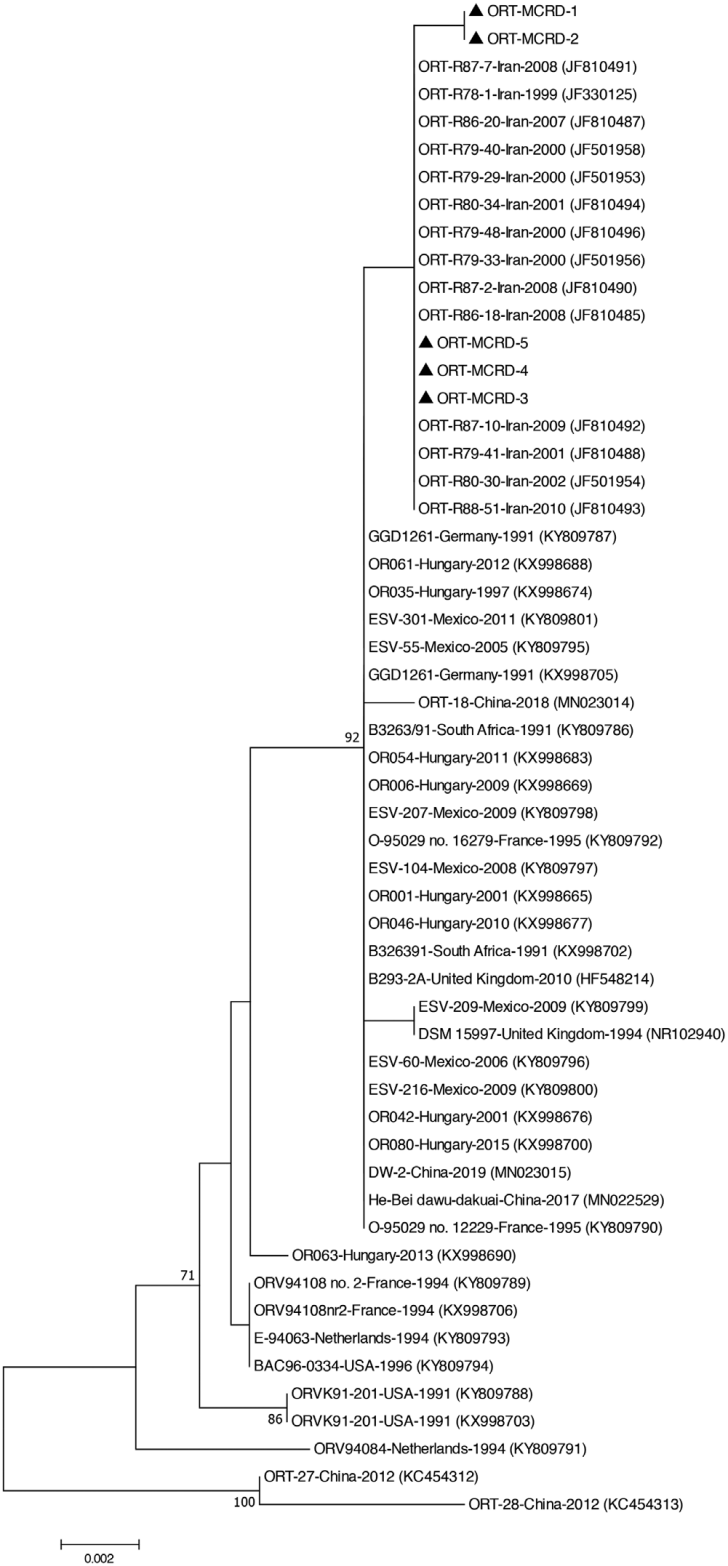
Phylogenetic relationships of the nucleotide sequences of *Ornithobacterium rhinotracheale* (ORT) strains based on the partial sequence of the 16S rRNA gene. The tree was generated by the neighbour‐joining method using the MEGA X software. The sequences from this study are indicated by the triangle‐shaped symbol.

The BLAST analysis of *mgc2*, *vlhA*, and 16S rRNA nucleotide sequences of studied bacteria is shown in Table 3. Five MG strains detected in this study displayed the highest nucleotide sequence identities to MG Iranian strains isolated in 2015–2016. Due to the limited number of complete sequences available in the database, two phylogenetic trees were constructed based on partial and complete coding sequences of *mgc2* genes (Figures 1 and 2). In both generated phylogenetic trees, all five MG strains segregated into distinct cluster along with other Iranian MG isolates. BLAST analysis revealed that the studied *vlhA* genes were most closely related to the Egyptian and Jordanian strains. In the phylogenetic tree, the two MS‐MCRD‐2 and MS‐MCRD‐3 bacteria were grouped into a disparate cluster together with MS strains isolated from Hungary, Austria, and Australia, while MS‐MCRD‐1 was clustered into a different branch along with the Jordanian strains isolated in 2013 (Figure 3). The BLAST search of the 16S rRNA genes showed that studied ORT strains were similar to the previously Iranian published sequences. Phylogenetic analysis revealed that 16S rRNA genes of ORT strains detected in this study were grouped into a cluster with other Iranian ORT isolates reported from 1999 to 2010 (Figure 4).

**TABLE 3:** Highest similarity of the *mgc2*, *vlhA*, and 16S rRNA genes in studied bacteria according to BLAST search.

Bacteria	Most similar to	Similarity (%)
MG‐MCRD‐1	Ck/Iran/MG‐46/16	100
	Ck/Iran/MG‐38/16	100
MG‐MCRD‐2	Ck/Iran/MG‐06/15	100
	Ck/Iran/MG‐46/16	99.78
MG‐MCRD‐3	Ck/Iran/MG‐46/16	100
	Ck/Iran/MG‐38/16	100
MG‐MCRD‐4	Ck/Iran/MG‐46/16	100
	Ck/Iran/MG‐38/16	100
MG‐MCRD‐5	Ck/Iran/MG‐46/16	100
	Ck/Iran/MG‐38/16	100
MS‐MCRD‐1	Ck/Jordan/IZSVE‐564‐D13‐3/13	93.32
	Tk/US/K1968/83	92.60
MS‐MCRD‐2	Ck/Egypt/SELIM‐CLEVB‐1/17	100
	Ck/Romania/MYCAV230/15	100
MS‐MCRD‐3	Ck/Egypt/SELIM‐CLEVB‐1/17	100
	Ck/Romania/MYCAV230/15	100
ORT‐MCRD‐1	Ck/Iran/ORT‐R88‐51/09	99.87
	Ck/Iran/ORT‐R87‐7/08	99.87
ORT‐MCRD‐2	Ck/Iran/ORT‐R88‐51/09	99.87
	Ck/Iran/ORT‐R87‐7/08	99.87
ORT‐MCRD‐3	Ck/Iran/ORT‐R87‐7/08	100
	Ck/Iran/ORT‐R87‐7/08	100
ORT‐MCRD‐4	Ck/Iran/ORT‐R88‐51/09	100
	Ck/Iran/ORT‐R87‐7/08	100
ORT‐MCRD‐5	Ck/Iran/ORT‐R87‐7/08	100
	Ck/Iran/ORT‐R87‐7/08	100

## 4 DISCUSSION

Upper respiratory tract infection is among the most common causes of high mortality rates and economic losses in global poultry industry. Respiratory diseases in poultry are caused by single infection or multiple infections of viral, bacterial, and fungal agents (Pantin‐Jackwood & Spackman, 2020). Despite numerous studies on respiratory viral agents in Iranian industrial poultry flocks (Bashashati et al., 2021; Haji‐Abdolvahab et al., 2019; Molouki et al., 2019), little information is available on the status of respiratory pathogenic bacteria in broiler flocks as part of the multiple respiratory infections. MG, MS, and ORT frequently co‐infect commercial poultry flocks with other respiratory pathogens. In the present study, we examined the role of MG, MS, and ORT in 70 broiler flocks suffering from respiratory disease using PCR. A total of five, three, and five flocks were found to be positive for MG, MS, and ORT, respectively. Compared to the results of another study in Algeria, all nine studied flocks were tested positive for MG, and MS was detected in only two flocks by PCR (Sid et al., 2015). The results were in agreement with the reports of Rahmani and Hosseini (2017), which indicated that avian mycoplasmas are not one of the main causes of respiratory diseases. The low detection rate of MG, MS, and ORT resulted from various factors in broiler farms: (a) the effectiveness of implemented control measures in breeder broiler farms (e.g., voluntary removal of infected flocks, vaccination of broiler breeder flocks with the MS‐H and inactivated ORT vaccines, and strict adherence to biosecurity practices on the farms) may account for the low detection rates of the studied bacteria in the current study, (b) use of anti‐mycoplasma drugs (including macrolides, pleuromutilins, and lincosamides) and other antibiotics for pathogenic bacteria in broiler farms decreases the chance of detecting MG, MS, and ORT, and (c) the sampling time significantly affects the isolation and detection of these bacteria. The reason is that the more time passes from the onset of the infection, the less chance there is to detect bacteria from the upper respiratory tract.

In respiratory diseases, the interaction of bacterial and viral agents can be either synergistic or antagonistic and might affect disease progression to varying degrees. These interactions, which can subsequently determine the severity of the disease complex, depend on various factors such as duration of interaction, host immune response, use of biological products (vaccine and drug), and environmental factors (Samy & Naguib, 2018). In addition to MG, MS, and ORT bacteria, other respiratory viruses such as AOaV‐1, ACV, and AIV subtype H9N2 were detected in the broiler flocks tested positive for studied bacterial respiratory pathogens. These viruses have been implicated by several authors as primary respiratory pathogens in Iranian commercial poultry farms suggesting that they play a prominent role in the clinical respiratory disease (Bashashati et al., 2021; Haji‐Abdolvahab et al., 2019; Molouki et al., 2019). Viral respiratory infections in birds cause tissue damage, accordingly facilitating the invasion of bacteria, resulting in loss of tissue function. Three possible mechanisms are considered for this tissue damage: (a) loss of cilia and ciliated cells as a result of virus replication in the upper respiratory tract, (b) inhibition of mucociliary clearance due to decreased ciliary activity, and (c) bacterial adherence and colonization due to damage to the epithelium (Bakaletz, 1995; El Ahmer et al., 1999; Wilson et al., 1996). On the contrary, a previous bacterial infection is beneficial for the pathogenesis of some viral agents such as low pathogenic AIV because it facilitates the cleavage of HA protein through protease‐producing bacteria (Kishida et al., 2004). However, bacterial infection may suppress viral pathogenesis by the modulation of the innate immune response and preventing virus entry to the susceptible cells (Sid et al., 2016). Avian mycoplasmas (MG and MS) often cause subclinical upper respiratory tract infections, and respiratory clinical signs are usually prominent in concomitant infection with other respiratory pathogens in poultry. Under field conditions, H9N2 viruses frequently coinfect other bacteria such as *E. coli*, MG, MS, ORT, *Avibacterium paragallinarum*, *Chlamydia psittaci*, and *Staphylococcus aureus* (Roussan et al., 2015; Samy & Naguib, 2018).

For molecular and phylogenetic analysis of MG, complete sequencing of the *mgc2* gene was performed based on a previous study (Bashashati & Banani, 2020). Mgc2 protein in different strains of MG differs in its sequence and number of amino acids. This variation is most noticeable in the proline‐rich region in its carboxyl terminus. This protein is responsible for binding to tracheal epithelial cells, and the coding sequence of this protein is often needed to differentiate MG strains (Ferguson‐Noel et al., 2005). In the current study, five MG strains were sequenced and compared with the MG sequences retrieved from the GenBank database. Based on complete and partial phylogenetic analysis of the *mgc2* gene, the detected MGs were grouped in the cluster of Iranian MG isolates from 2015 to 2016 (Bashashati & Banani, 2020). In a study conducted in Iran, 10 MG isolates were genetically analyzed based on complete sequencing of *mgc2* and *pvpA* genes. The results of the phylogenetic analysis showed that the Iranian strains isolated from 2015 to 2016 were clustered into two distinct branches. The three isolates were grouped with S6 strain as well as Egyptian and Pakistani isolates, and the other seven MGs formed a disparate group with other isolates from Thailand and Italy (Bashashati & Banani, 2020). According to partial sequencing of the *pvpA* gene, MG strains from Russia, the United States, Australia, China, and Iran were divided in three clusters, namely, I, II, and III. Group I represented most Russian isolates, and the Iranian MG formed a branch (group II) together with the US isolate and R strain. In group III, other Russian MGs were clustered along with isolates from the US as well as 6/85, S6, ts‐11, and F strains (Sprygin et al., 2010). In gene‐targeted sequencing, the US MGs isolated from poultry and house finches clustered with vaccine strains. The other US and Australian isolates formed another group, while the Israeli strains clustered in a disparate cluster (Ferguson‐Noel et al., 2005). According to the results obtained, it can be concluded that Iranian MGs are distinct from those found in other countries and vaccine strains.

Given that the live attenuated vaccine MS‐H has been used in Iranian breeder flocks since 2005, the differentiation of field and vaccine strains is crucial. Bayatzadeh et al. investigated 43 broiler flocks regarding MS infection from 2001 to 2003. About 60% of oropharyngeal swabs were reported to be positive for the 16S rRNA gene using the PCR technique (Bayatzadeh et al., 2011). In a study, of 21 broiler farms, eight flocks tested positive using MS specific primer targeting the 16S rRNA and *vlhA* genes. MS was confirmed in two apparently healthy broiler flocks, indicating the role of MS in subclinical infections (Ghaniei, 2016). A total of 70 broiler flocks with the history of respiratory disease were tested in this study, of which three samples were established to be MS‐positive. Despite the vaccination of breeder flocks with the MS‐H vaccine, the MS isolates were different from the vaccine strain in their progeny. The results of this study were in agreement with those of previous studies (Bayatzadeh et al., 2014; Ghaniei, 2016). Phylogenetic analysis indicated that the two MS samples detected were clustered into a group together with strains from Europe and Australia. In contrast, one of the strains shared an out‐group relationship with the Jordanian isolates. According to the previous studies, the MS strains were genetically distinct from isolates of non‐Iranian origin (Bayatzadeh et al., 2014; Ghaniei, 2016).

Ghodsian et al. showed that Iranian ORT strains formed a separate cluster using the partial sequencing of the 16S rRNA gene. The average nucleotide homology of the 10 ORT isolates with each other is higher (100%) compared to other strains (98.60%) isolated from different parts of the world (Ghodsian et al., 2013). Hassanzadeh et al. isolated and detected four ORT strains from broilers and compared them with other isolates through the partial sequencing of the 16S rRNA gene. Nucleotide homology of more than 98% was observed between four studied isolates and nine strains obtained from GenBank (Hassanzadeh et al., 2010). Mirzaei et al. indicated a high similarity of the 16S rRNA gene sequences (98%–100%) of ORT isolates from pigeon, quail, and turkey, and other reference sequences were retrieved from GenBank. The phylogenetic tree showed that the Iranian pigeon ORT isolates formed a separate cluster from other ORTs isolated from broilers (Mirzaie et al., 2011). In the present study, the studied ORT strains were highly similar (about 100%) and clustered with other Iranian isolates.

## 5 CONCLUSION

This study has shown that chickens with respiratory clinical signs could harboured multiple chicken respiratory pathogens. Although a low detection rate of MG, MS, and ORT in the multiple respiratory infections was observed, further molecular analyses with larger sample sizes are crucial to determine the epidemiological characteristics of these bacteria and to outline strategies to minimize them in the poultry farms. In addition, whole‐genome sequencing or other bacterial typing methods are useful to distinguish the relationship of bacterial genotypes to other strains and trace the source of infection.

## AUTHOR CONTRIBUTIONS

Conceptualization, data curation, formal analysis, funding acquisition, investigation, methodology, project administration, resources, supervision, writing—original draft, and writing—review and editing: Mohsen Bashashati. *Formal analysis, methodology, and writing—review and editing*: Mohammad Shojaei. *Conceptualization, Methodology, Writing—original draft, and Writing—review and editing*: Fereshteh Sabouri.

## CONFLICT OF INTEREST STATEMENT

The authors declare no conflict of interest.

### ETHICS STATEMENT

Ethics approval was not needed because this article did not contain any studies with animals performed by any of the authors.

### PEER REVIEW

The peer review history for this article is available at https://publons.com/publon/10.1002/vms3.1162.

## Data Availability

The datasets generated during and/or analyzed during the current study are available from the corresponding author on reasonable request.
